# Predicting the Thrill: How Individual and Environmental Factors Shape Thrilling Perceptions of Violent and Non-violent Crime

**DOI:** 10.1007/s40865-025-00276-7

**Published:** 2025-10-29

**Authors:** Curtis D. Smith, Cortney Simmons, Emma Rodgers, Elizabeth Cauffman

**Affiliations:** 1 Department of Psychological Science, School of Social Ecology, University of California, Irvine, 4220 Social and Behavioral Sciences Gateway, 214 Pereira Dr, Irvine, CA 92617 USA; 2 School of Interdisciplinary Forensics, Arizona State University, Tempe, AZ USA

**Keywords:** Thrill of crime, Perceived rewards, Juvenile justice, Adolescents, Thrill-seeking

## Abstract

This study longitudinally investigates how individual and environmental factors predict thrilling perceptions of criminal behavior using a sample of 1009 justice-involved youth (ages 15–21) who were followed over 7 years. Mixed effect regression models indicated that peer delinquency, youth offending, and impulsive-irresponsible traits were associated with thrilling perceptions of both violent and non-violent crime. Comparatively, callous-unemotional traits predicted only the thrill of violent crime but not non-violent crime. Additionally, the association between peer delinquency and the thrill of non-violent crime waned as participants aged, suggesting a decreased susceptibility to peer influence over time. These individual differences, paired with these socially transmitted beliefs from peers, highlight how thrilling perceptions of crime may be reinforced by youth’s characteristics or vicariously through their peers. However, while youth may age out of susceptibility to peer influence, these other influences sustained across age. These findings advance our understanding of the persistence and influence of adolescents’ experiences in shaping thrilling perceptions of crime, hinting at potential mechanisms that contribute to antisocial behavior.

While frequently perceived as adverse and antisocial, some may find engagement in criminal behavior appealing. Despite the myriad negative consequences of engaging in crime, some aspects may be personally rewarding. For example, individuals may be motivated to engage in crime for money, power, revenge, or even their survival (Coleman, 1992; Kivivuori et al., 2015; Tyler & Johnson, 2004). Early theorists proposed that in addition to these potential rewards, some individuals may commit criminal acts simply because they find them thrilling or enjoyable (Frazier & Meisenhelder, 1985; Katz, 1988). Indeed, extant literature confirms that individuals are more likely to engage in crimes they view as intrinsically rewarding (Thomas et al., 2020; Wojciechowski, 2020). An effect that varies by age, with adolescents being the most susceptible to this pattern (Altikriti et al., 2022, 2023). Thus, the present study specifically focuses on the thrill of crime, which encompasses how fun or exciting adolescents perceive criminal conduct to be.

Adolescents are more oriented toward rewarding experiences than adults, gravitating toward high-risk and high-reward scenarios (Barkley-Levenson & Galván, 2014; Braams et al., 2015; Cservenka et al., 2013). Thus, the perceived benefits of behavior outweigh the consequences (Loughran et al., 2016). Perceiving any benefit from crime is criminogenic (Baker & Piquero, 2010), but thrilling perceptions place adolescents at exceptional risk due to their heightened reward sensitivity. Concurrently, sensation seeking also peaks during adolescence (Cauffman et al., 2010; Steinberg et al., 2017; Steinberg, 2007) and has been linked to higher rates of risk-taking (Burt & Simons, 2013; Galván, 2013; Harden et al., 2011; Lynam & Miller, 2004; Shulman et al., 2017; Steinberg, 2008). However, sensation seeking reflects a broad, generalized interest in novel, intense experiences (Zuckerman, 2009). Those high in sensation seeking are just as much at risk for thrilling perceptions of positive behaviors (i.e., playing sports, riding rollercoasters) as they are for negative behaviors (i.e., crime; Duell & Steinberg, 2018; Fryt et al., 2021). The thrill of crime is an individual’s perception of criminal behavior, specifically, as being fun or exciting. This perception gauges how sensational the experience of crime is perceived rather than one’s general propensity to be sensation seeking.

Prior research on the perceived thrill of crime has examined the contributions of both contextual and individual factors in forming youths’ understanding of crime (Altikriti et al., 2022; Trinidad et al., 2018). According to Schmidt et al. (2021), youth make meaning of and adopt their perceptions of crime from their context, whether that be the people, experiences, or physical properties embedded within these systems. Additionally, certain individual characteristics and behaviors also shape thrilling perceptions of crime, increasing one’s likelihood to find antisocial behavior thrilling.

## Contextual Factors

### Neighborhood Disorder & Exposure to Violence

The social and physical composition of neighborhoods can significantly affect youths’ perceptions of criminal behavior. Highly disadvantaged and disorganized communities have been found to have increased instances of gang activity and violence (Butcher et al., 2015; Gibson et al., 2009; Patchin et al., 2006), which can foster an approving culture of criminal behavior (Stewart & Simons, 2010). In addition, adolescents within the justice system report violence exposure at disproportionately higher rates before and during system involvement when compared to uninvolved adolescents (Dierkhising et al., 2013; Baglivio et al., 2014). These violent experiences may impact thrilling perceptions of crime. For example, Alwood and Bell (2008) found that violence-exposed youth become ensnared in a culture of violence that indoctrinates positive beliefs regarding violence proactively or reactively. Research has similarly observed greater endorsement of pro-violence attitudes in youth who report greater violence exposure (Muradwij & Allwood, 2021; Slovak et al., 2007). Adverse neighborhood conditions and exposure to violence during adolescence can also normalize violent behavior (Esposito et al., 2022). Several studies assert that these broader contextual factors contribute to youth developing thrilling perceptions of antisocial behavior as a result (Ching et al., 2012; Thomas et al., 2022). On the contrary, some youth become more averse to violence given these adverse early experiences (Jain & Cohen, 2013). Furthermore, recent literature has observed resilience among those exposed to violence (Wright et al., 2016). Thus, it is possible that adolescents in these contexts could also find crime less thrilling due to these adverse experiences.

### Peer Delinquency

Peer relationships are consistently linked with adolescent attitudes and behaviors; having peers who engage in delinquency contributes to delinquent behavior in adolescence (Gillespie et al., 2022). Adolescents’ perceptions of both the risks and rewards of antisocial behaviors (e.g., crime, substance use) are similarly impacted by the beliefs held by their peers (Albert et al., 2013; Hoeben & Thomas, 2019). Megens and Weerman (2011) observed that having peers with more pro-criminal attitudes increased the likelihood of youth endorsing similarly valenced attitudes, suggesting a transference of beliefs to adolescents from their peers. Involvement with peer groups that endorse crime as a thrilling experience may encourage the adoption of similar perceptions. Indeed, previous findings indicate that the mere presence of peers further sensitizes adolescents to rewards (Albert et al., 2013; Chein et al., 2010). Brezina and Piquero (2003) emphasized that adolescents actually report finding crime more thrilling when accompanied by their peers. Thus, the significance of peer influence, as a whole, may in turn shape adolescents’ thrilling perceptions of crime.

## Individual Factors

### Psychopathic Traits

At the individual level, psychopathy is a robust prospective risk factor for future criminal behavior, and recent investigations emphasize the role of perceived benefits of crime in this association (Prospero-Luis et al., 2017). Psychopathy is a multidimensional construct characterized by several traits indicative of disinhibition and socioemotional difficulties (Colins et al., 2016; Hare, 2006). Researchers have organized these traits into three dimensions: grandiose manipulative traits (i.e., narcissism and dishonest charm), impulsive-irresponsible traits (i.e., thrill seeking and impulsivity), and callous-unemotional (CU) traits (i.e., apathy and emotional inexpression). Prior literature suggests that system-involved adolescents who endorse higher psychopathic traits also are more likely to report perceiving crime as thrilling (Altikriti & Nedelec, 2020; Ray et al., 2020). Prospero-Luis et al. (2017) found that the callous-unemotional and impulsive-irresponsible dimensions of psychopathy influenced the thrill of crime; however, they neglected to parse out the thrill of violent versus non-violent crimes. Grandiosity and manipulativeness are commonly observed among those who commit non-violent offenses (Merzagora et al., 2014; Perri, 2011), suggesting that specific facets of psychopathy may differentially influence thrilling perceptions depending on the type of crime.

### Offending

An individual’s own offending behavior may also serve as a relevant contributing factor to thrilling perceptions of crime, as perceptions of behaviors are driven in part by our own experiences (Snyder et al., 2015). Specifically, it is possible that a positive offending experience (e.g., stealing money without getting caught) may in turn amplify the thrilling perceptions of committing that offense. Loughran et al. (2009) conducted a longitudinal evaluation of perceived risks and rewards of offending amongst system-involved adolescents and found that those who offend more perceived crime to be more thrilling than those with less experience. Other researchers have found similar results of prior offending being linked to a greater perceived thrill of crime (Altikriti & Nedelec, 2020; Shulman, 2017), offering further support that system-involved adolescents’ level of engagement in offending may contribute to how thrilling they perceive it to be.

## Crime Typology

In 2020, only 8% of youth arrests were for violent crimes such as murder, assault, or robbery (Puzzanchera, 2022). Non-violent crimes such as shoplifting or vandalism account for the vast majority of crimes among youth. Unlike violent crimes, non-violent crimes do not require physical aggression or harm toward another person to commit the act. Youth have been shown to perceive these crimes as less serious compared to adults (Bensimon & Bodner, 2012). Furthermore, most youth report engaging in criminal acts like vandalism, shoplifting, or auto theft for fun (Loughran et al., 2016; Lopez, 2008; Jacobs & Cherbonneau, 2022). These findings suggest that finding non-violent crime thrilling is more common compared to violent crime. Therefore, their antecedents may also differ.

## Developmental Considerations

Importantly, potential age-related trends in the progression of the perceived thrill of crime have not been explored in previous literature despite the knowledge that patterns of criminal behavior change across adolescence (Moffit, 1993; Sweeten et al., 2013). Additionally, extant literature on system-involved adolescents highlights how their developing capacities (e.g., psychosocial maturity, cognitive control, and impulsivity) change in early adolescence compared to later adolescence (Icenogle et al., 2019). The salience of contextual and individual risk (e.g., violence exposure or having peers who engage in delinquency) may vary at specific ages, potentially exhibiting a more significant impact at earlier or later ages. For example, previous research suggests that adolescents become less susceptible to their environment as they age, potentially due to their capacity to exercise more control over it (Dey & Pierret, 2014). One of the most robust findings in criminological literature is that offending behavior increases in adolescence and decreases thereafter (Hirschi & Gottfredson, 1983; Piquero et al., 2003; Sweeten et al., 2013). Examining these influences by age may reveal how developmentally sensitive the risk factors for the thrill of crime may be.

## The Current Study

The present study aims to examine whether several individual and contextual risk factors were associated with thrilling perceptions of violent and non-violent crime throughout adolescence within a sample of system-involved male adolescents. Risk factors were examined simultaneously to identify the strongest determinants of thrilling perceptions and whether they differed by violent or non-violent crime. We expect peer delinquency, impulsive-irresponsibility, and neighborhood disorder to be associated with the thrill of crime, whether violent or non-violent. However, we expect callous-unemotional traits and violence exposure to be uniquely predictive of the thrill of violent crime and grandiose-manipulativeness to predict the thrill of non-violent crime. This sample is well-suited to address this aim, given that males are more prone to sensation seeking (Cross et al., 2013) and have a higher propensity for thrill seeking during adolescence (Shulman et al., 2014). Further, the longitudinal study design permits the use of mixed effect modeling to examine how the thrill of crime changes with age and whether the associations between the proposed predictors of the thrill of crime change across adolescence, allowing for the identification of potential sensitive periods.

## Method

Data for this study were drawn from the longitudinal Pathways to Desistance study (Mulvey et al., 2004; Schubert et al., 2004). The sample consisted of 1354 male youth from Maricopa County, AZ, and Philadelphia County, PA, who were recruited after being found guilty of a felony offense or serious misdemeanor such as robbery or aggravated assault. To be eligible for the study, individuals had to reside in Maricopa County, AZ, or Philadelphia, PA, be found guilty of a serious offense, and be between the ages of 14 and 18 at the time of adjudication. Informed parental consent and youth assent were attained before study initiation and before each interview. Youth completed a baseline interview after their adjudication hearing (between November 2000 and March 2003). Follow-up interviews were completed every 6 months for three years and annually for an additional four years thereafter (the first follow-up interview was completed in May 2001; the last in March 2010). Sample retention was high (range = 84–94%, *M* = 90%). Trained research assistants administered questionnaires through computer-assisted interviews that took place in a location convenient for the participants (e.g., participants’ homes, public places, secure detention, and other residential facilities). For questions about sensitive material (e.g., criminal behavior, substance use), answers were provided using a portable keypad to ensure confidentiality. Adolescents were informed that the study team received a Privacy Certificate set by the U.S. Department of Justice that prohibited disclosure of information to anyone outside the research staff, except in cases of participants expressing that they are being harmed, intend to harm someone else, or intend to harm themselves. Adolescents were paid $50 for their participation in initial interviews, and payments increased at each subsequent interview to encourage sample retention. All procedures were approved by the institutional review boards at Arizona State University, Temple University, and the University of Pittsburgh. For further information regarding the enrollment process, study procedures, and sample characteristics, see Schubert and colleagues (2004). These data are publicly available within the Inter-university Consortium for Political and Social Research.

### Measures

#### Thrill of Violent and Non-violent Crime

The Personal Rewards of Crime subscale from the Indices of Personal and Social Costs and Rewards measure (Nagin & Paternoster, 1994) was used to assess the youths’ perceived thrill of crime. The 7-item self-report subscale assessed how much of a “thrill or rush” youth perceive when they engage in violent crime (e.g., fighting, armed robbery, stabbing someone) and non-violent crime (e.g., breaking into a home or store, stealing from a store, vandalism, auto theft). Youth responded on a Likert scale ranging from 0 (no fun or kick at all) to 10 (a great deal of fun for each item). A thrill of violent crime score was computed by summing the violent items, and a thrill of non-violent crime score was computed by summing the non-violent items. The violent (α =.78 to.83) and non-violent (range, α =.85 to.92) scores showed acceptable to good internal consistency across each timepoint. Due to skewness, both variables were log-transformed.

#### Victimization and Witnessing Violence

The Exposure to Violence Inventory (Selner‐O’Hagan et al., 1998) was used to assess exposure to violent events. Six binary items assessed whether the youth was a victim of violence (e.g., “Have you been chased where you thought you might be seriously hurt in the past six months?”), and seven binary items assessed whether the youth witnessed violence (e.g., “Have you seen someone else being raped, an attempt made to rape someone or any other type of sexual attack in the past six months?”). A victimization score was computed by summing the victimization items (range, α =.51 to.62), and a witnessing violence score was computed by summing the witnessing violence items (range, α =.71 to.78).

#### Neighborhood Disorder

During baseline and each of the follow-up interviews, the Neighborhood Conditions Measure (Sampson & Raudenbush, 1999) was used to estimate disorder in the environment surrounding the youth’s home. The 21-item self-report measure assessed physical disorder (e.g., “cigarettes on the street or in the gutters,” “graffiti or tags”) and social disorder (e.g., “adults fighting or arguing loudly,” “people using needles or syringes to take drugs”) in the neighborhood. Youth responded on a 4-point Likert scale ranging from 1 (“Never”) to 4 (“Often”). A total neighborhood disorder score was computed by averaging the physical and social disorder items. The neighborhood disorder scores showed good internal consistency at each time point (α =.96).

#### Peer Delinquency

During baseline and each of the follow-up interviews, the Peer Delinquent Behavior measure (Thornberry et al., 1994) was used to assess peer delinquent behavior. The 12-item self-report scale assessed the prevalence of friends who engage in delinquent behaviors (e.g., “During the recall period, how many of your friends have sold drugs?”). Youth responded on a 5-point Likert scale ranging from 1 (“None of them”) to 5 (“All of them”). The items were summed to generate a peer delinquent behavior score. The score showed good internal consistency across each time point (range, α =.87 to.90).

#### Psychopathic Traits

During each of the follow-up interviews, the Youth Psychopathic Traits Inventory (YPI; Andershed et al., 2002) was used to assess the Impulsive-Irresponsible, Callous-Unemotional, and Grandiose-Manipulative dimensions of psychopathic traits in youth. The 15-item self-report Impulsive-Irresponsible subscale assessed sensation seeking (e.g., “I like to be where exciting things happen”), impulsiveness (e.g., “I consider myself as a pretty impulsive person”), and irresponsibility (e.g., “I have often been late to work or classes in school”). The 15-item self-report Callous-Unemotional subscale assessed remorselessness (e.g., “To feel guilt and regret when you have done something wrong is a waste of time”), unemotionality (e.g., “I usually feel calm when other people are scared”), and callousness (e.g., “I think that crying is a sign of weakness, even if no one sees you”). The 20-item self-report Grandiose-Manipulative subscale assessed the interpersonal traits, which includes traits such as dishonest charm (e.g., “I have the ability to con people by using my charm and smile”), grandiosity (e.g., “I’m better than everyone on almost everything”), lying (e.g., “Sometimes I lie for no reason, other than because it’s fun”), and manipulation (e.g., “I can make people believe almost anything”). Youth responded on a 4-point Likert scale ranging from 1 (“Does not apply at all”) to 4 (“Applies very well”). Several positively worded items in the subscales were reverse coded. An Impulsive-Irresponsible traits score was computed by summing the thrill seeking, impulsiveness, and irresponsibility items, a Callous-Unemotional score was computed by summing the remorselessness, unemotionality, and callousness items, and Grandiose-Manipulative traits score was computed by summing the dishonest charm, grandiosity, lying, and manipulation items. The Impulsive-Irresponsible (α =.82 to.87), Callous-Unemotional (range, α =.73 to.79), and Grandiose-Manipulative (α =.91 to.92) scores showed acceptable to good internal consistency across each timepoint.

#### Violent and Non-violent Offending

During baseline and each of the follow-up interviews, the Self-Reported Offending measure (Huizinga et al., 1991) was used to evaluate youth involvement in antisocial and illegal activities. The 24-item measure assessed involvement in violent (e.g., been in a fight, shot at someone) and non-violent acts (e.g., entered a building to steal, drove drunk or high). Youth indicated whether they had engaged in each act at least once (0 = No, 1 = Yes). A violent offending score was computed by summing all violent items (α =.74), and a non-violent offending score was computed by summing all non-violent items (α =.80).

### Demographics

Several demographic covariates were modeled separately and within the full model to account for any potential demographic differences that may influence our outcomes. These included participants’ self-identified race or ethnicity (coded as White, Black, Hispanic, and Other Racial Category/Multiracial). Parental education was also included and has been validated as a proxy for socioeconomic status in prior research in adolescent samples (Lien et al., 2001), with higher scores indicating lower socioeconomic status.

### Analytic Plan

Data from the 6-, 12-, 18-, 24-, 30-, 36-, 48-, and 60-month follow-up interviews were used in the analyses. Because we were interested in how the perceived thrill of crime and the predictors of it changed across adolescence (instead of how they changed from the first interview), the data were restructured to be aligned by participant age rather than by interview, with the final data set resembling an overlapping cohort design. When necessary, six-month interviews conducted within an annual year were combined to be consistent with the recall period of the annual assessments. The age range in the resulting restructured data set was 14.5 years old to 24.5 years old. The final age range was restricted to 15 to 21 years old due to the small sample sizes at the upper and lower tails of the age range. The final analytic sample included 1009 (40% Black, 34% Hispanic, 22% White, 4% Other) youth between the ages of 14 and 19 (*M* = 16.30, *SD* = 1.16) at recruitment.

Mixed effect regression models estimated in Stata v17 (StataCorp) were used to investigate associations between the perceived thrill of crime, violence exposure, and other individual and environmental risk factors. Mixed effects models accommodate missing data using conditional maximum likelihood estimation, which incorporates all available information to generate model estimates rather than relying on complete case analysis. Our analyses were conducted in five steps. First, we examined how the perceived thrill of violent and non-violent crime changed across age, which was treated as a continuous predictor. Second, we examined the associations between the thrill of crime and the environmental risk factors (victimization, witnessing violence, neighborhood disorder, and peer delinquent behavior), individual risk factors (offending and impulsive-irresponsible, callous-unemotional, and grandiose-manipulative traits), and demographic covariates (race/ethnicity, parent education) separately and together. The thrill of crime and the individual/contextual predictors were concurrent. Finally, we examined whether any of the associations between the thrill of crime and the contextual and individual risk factors varied across adolescence by testing two-way interaction terms between age and each individual and contextual predictor. The interaction terms were tested in separate models, adjusting for the main effects of the other predictors.

### Missing Data

Regressions were conducted to determine whether having any missing data was associated with the study variables at each age. Youth with missing data reported less violent offending at age 15 (b = −1.22, SE = 0.07, *p* < 0.001), age 16 (b = −0.96, SE = 0.07, *p* < 0.001), age 17 (b = −0.53, SE = 0.09, *p* < 0.001), and age 18 (b = −0.22, SE = 0.11, *p* = 0.05). Youth with missing data also reported less non-violent offending at age 15 (b = −0.53, SE = 0.13, *p* < 0.001), age 16 (b = −1.32, SE = 0.10, *p* < 0.001), age 17 (b = −1.02, SE = 0.12, *p* < 0.001), age 18 (b = −0.88, SE = 0.16, *p* < 0.001), age 19 (b = −0.53, SE = 0.13, *p* < 0.001), and age 20 (b = −0.45, SE = 0.12, *p* < 0.001). Having missing data was not associated with the perceived thrill of violent or non-violent crime, callous-unemotional traits, impulsive-irresponsible traits, grandiose-manipulative traits, victimization, witnessing violence, neighborhood disorder, or peer delinquency at any age. Given these results, we believe it is unlikely that the associations discovered in the present analyses were substantively impacted by missing data.

## Results

### Descriptive Statistics

Descriptive information of study variables at each age is presented in Table 1, and a bivariate correlation matrix of study variables is presented in Table 2.

**Table 1:** Descriptive statistics for main study variables by participant age

	Age 15 *n*=156 M(SD)	Age 16 *n*=339 M(SD)	Age 17 *n*=556 M(SD)	Age 18 *n*=767 M(SD)	Age 19 *n*=719 M(SD)	Age 20 *n*=224 M(SD)	Age 21 *N*=64 M(SD)
Continuous Variable							
Thrill of Violent Crime	2.48	2.69	2.57	2.43	2.16	1.97	1.69
	(2.64)	(2.62)	(2.49)	(2.60)	(2.45)	(2.35)	(2.13)
Thrill of Non-violent Crime	2.33	2.18	1.92	1.71	1.48	1.42	1.15
	(2.62)	(2.52)	(2.29)	(2.36)	(2.22)	(2.29)	(1.87)
Victimization	.34	.39	.32	.37	.29	.25	.22
	(.80)	(.78)	(.70)	(.76)	(.68)	(.63)	(.68)
Witnessing Violence	1.38	1.44	1.25	1.41	1.23	1.04	.75
	(1.73)	(1.67)	(1.56)	(1.68)	(1.67)	(1.46)	(1.36)
Neighborhood Disorder	2.32	2.35	2.32	2.35	2.34	2.35	2.35
	(.76)	(.77)	(.78)	(.79)	(.81)	(.79)	(.83)
Peer Delinquency	1.84	1.92	1.82	1.85	1.76	1.69	1.57
	(.81)	(.80)	(.74)	(.75)	(.72)	(.70)	(.68)
Impulsive-Irresponsibility	35.32	35.03	34.52	34.36	33.59	32.98	32.48
	(7.97)	(8.07)	(7.60)	(7.73)	(8.00)	(8.05)	(8.97)
Callous-Unemotional	33.14	33.23	32.83	32.76	32.27	32.73	32.48
	(6.42)	(6.19)	(5.65)	(6.43)	(6.19)	(6.34)	(6.27)
Grandiose-Manipulative	39.81	39.60	38.93	39.02	37.52	37.74	37.77
	(10.97)	(10.72)	(10.48)	(10.57)	(10.71)	(10.43)	(10.71)
Violent Offending	1.30	1.19	1.13	1.02	.762	.53	.47
	(1.78)	(1.47)	(1.52)	(1.54)	(1.24)	(1.02)	(.73)
Non-violent Offending	1.57	1.49	1.41	1.48	1.24	1.10	.53
	(2.66)	(2.33)	(2.19)	(2.30)	(2.03)	(1.93)	(1.01)

*M*=Mean. *SD*=Standard Deviation

**Table 2:** Correlations between main study variables

	1	2	3	4	5	6	7	8	9	10	11
1. Thrill of Violent Crime	-										
2. Thrill of NV Crime	.645*	-									
3. Victimization	.199*	.169*	-								
4. Witnessing Violence	.167*	.137*	.486*	-							
5. Neighborhood Disorder	-.084*	-.062*	.134*	.280*	-						
6. Peer Delinquency	.327*	.321*	.364*	.462*	.240*	-					
7. Impulsive-Irresponsible	.363*	.397*	.210*	.198*	.037*	.397*	-				
8. Callous-Unemotional	.340*	.327*	.159*	.207*	.080*	.350*	.622	-			
9. Grandiose-Manipulative	.273*	.309*	.164*	.170*	.057*	.326*	.692*	.685*	-		
10. Violent Offending	.343*	.296*	.491*	.504*	.135*	.518*	.324*	.296*	.225*	-	
11. NV Offending	.284*	.314*	.438*	.465*	.145*	.559*	.367*	.321*	.276*	.716*	-

*NV*=Non-violent. * indicates significant correlations (p<.05)

### Thrill of Non-Violent Crime

Mixed effect regression models were conducted in a stepwise fashion to examine the effects of various factors on thrilling perceptions of non-violent crime. Results indicated that the thrill of non-violent crime was not associated with youths’ age (see Table 3, Model 1). When examining contextual factors, neighborhood disorder and peer delinquency were the only significant factors, such that less disordered neighborhoods and greater peer delinquency were associated with greater thrilling perceptions of non-violent crime (see Table 3, Model 2). Impulsive and irresponsible traits and offending were the only significant individual factors, such that higher scores were associated with greater thrilling perceptions of non-violent crime (see Table 3, Model 3). While not of substantive interest, each demographic covariate was significant, such that being White or Hispanic (relative to Black) was associated with greater thrilling perceptions of non-violent crime (see Table 3, Model 4). In the full model, with all factors examined simultaneously, peer delinquency, impulsive-irresponsible traits, and non-violent offending were the only main study variables significantly associated with the thrill of non-violent crime, such that higher scores on each were associated with increased thrill (see Table 3, Model 5).

**Table 3:** Mixed effects regression models estimating associations between risk factors and the thrill of non-violent crime

	Model 1 Age			Model 2 Contextual Factors			Model 3 Individual Factors			Model 4 Demographics			Model 5 Full		
	*B*	*p*	*95% CI*	*B*	*p*	*95% CI*	*B*	*p*	*95% CI*	*B*	*p*	*95% CI*	*B*	*p*	*95% CI*
Age	−0.026	0.110	−0.057, 0.006										−0.016	0.306	−0.046, 0.015
Victimization				0.048	0.122	−0.013, 0.108							0.018	0.561	−0.043, 0.079
Witnessing Violence				0.024	0.146	−0.056, 0.008							−0.032	0.051	−0.064, 0.000
Neighborhood Disorder				−0.095	0.006	−0.162, −0.027							−0.052	0.148	−0.122, 0.018
Peer Delinquency				0.197	0.000	0.136, 0.258							0.081	0.019	0.014, 0.148
Impulsive-Irresponsible							0.023	0.000	0.014, 0.032				0.018	0.000	0.009, 0.027
Callous-Unemotional							0.005	0.317	−0.005, 0.015				0.005	0.290	−0.005, 0.016
Grandiose-Manipulative							0.002	0.506	−0.004, 0.008				0.003	0.290	−0.003, 0.010
Offending							0.030	0.000	0.014, 0.046				0.027	0.007	0.008, 0.047
White v. Black										0.315	0.000	0.163, 0.467	0.189	0.019	0.031, 0.348
Hispanic v. Black										0.436	0.000	0.290, 0.583	0.307	0.000	0.157, 0.458
Other v. Black										0.259	0.059	−0.010, 0.528	0.144	0.288	−0.121, 0.409
Parent Education										-.029	0.388	−0.095, 0.037	−0.014	0.661	−0.078, 0.050

All models adjusted for time

**Fig. 1:**
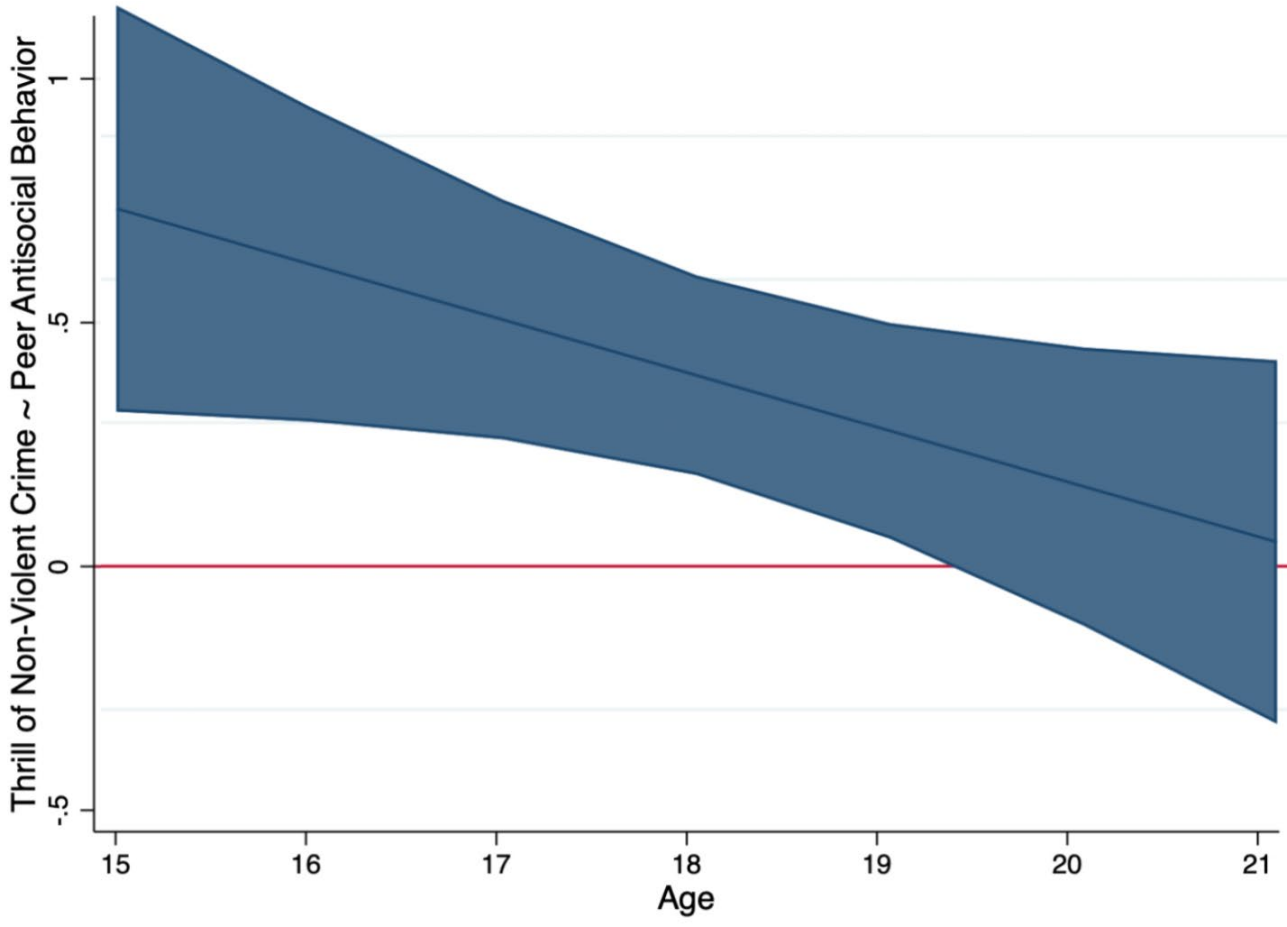
The predicted regression coefficient (solid blue line) and 95% CI band for peer delinquency by age

Significant interaction terms between each risk factor and age were then added to the full model individually to test for any potential age interactions. A significant two-way interaction was only observed for the association between thrill of non-violent crime and peer delinquency (see Table 4), such that this peer delinquency was only significantly associated with the thrill of non-violent crime between the ages of 15 and 18 years old (see Table 5; Fig. 1).

**Table 4:** Probed interaction term between peer delinquent behavior and age predicting the thrill of non-violent crime

	Thrill of Non-violent Crime X Peer Deviancy		
	*B*	*p*	*95% CI*
Age			
15	0.183	0.002	0.066, 0.300
16	0.143	0.002	0.053, 0.234
17	0.104	0.004	0.033, 0.175
18	0.065	0.054	−0.001, 0.131
19	0.025	0.520	−0.052, 0.103
20	−0.014	0.788	−0.114, 0.087
21	−0.053	0.419	−0.182, 0.076

All models adjusted for time

**Table 5:** Mixed effects regression models estimating associations between risk factors and the thrill of violent crime

	Model 1 Age			Model 2 Contextual Factors			Model 3 Individual Factors			Model 4 Demographics			Model 5 Full		
	*B*	*p*	*95% CI*	*B*	*p*	*95% CI*	*B*	*p*	*95% CI*	* B*	*p*	*95% CI*	*B*	*p*	*95% CI*
Age	0.016	0.232	−0.010, 0.042										0.032	0.014	0.007, 0.058
Victimization				0.038	0.128	−0.011, 0.087							0.016	0.527	−0.034, 0.066
Witnessing Violence				0.023	0.081	−0.003, 0.049							0.016	0.256	−0.011, 0.042
Neighborhood Disorder				−0.077	0.008	−0.134, −0.020							−0.032	0.279	−0.091, 0.026
Peer Delinquency				0.212	0.000	0.155, 0.169							0.134	0.000	0.075, 0.193
Impulsive-Irresponsible							0.014	0.000	0.006, 0.021				0.009	0.022	0.001, 0.016
Callous-Unemotional							0.019	0.000	0.011, 0.027				0.018	0.000	0.010, 0.026
Grandiose-Manipulative							0.000	0.879	−0.005, 0.004				0.000	0.903	−0.004, 0.005
Offending							0.058	0.000	0.035, 0.081				0.031	0.028	0.003, 0.058
White v. Black										0.214	0.002	0.079, 0.349	0.191	0.006	0.054, −0.422
Hispanic v. Black										0.348	0.000	0.225, 0.472	0.301	0.000	0.179, 0.422
Other v. Black										0.246	0.042	0.009, 0.482	0.181	0.102	−0.036, 0.398
Parent Education										-.096	0.001	−0.154, −0.038	−0.081	0.003	−0.136, −0.027

All models adjusted for time

### Thrill of Violent Crime

Mixed effect regression models were conducted in a stepwise fashion to examine the effects of various factors on thrilling perceptions of violent crime. Results indicated that the thrill of non-violent crime was not associated with youths’ age (see Table 6, Model 1). When examining contextual factors, neighborhood disorder and peer delinquency were the only significant factors, such that less disordered neighborhoods and greater peer delinquency were associated with greater thrilling perceptions of violent crime (see Table 6, Model 2). Several individual risk factors were significant, including CU traits, offending, and impulsive-irresponsible traits, such that higher scores were associated with greater thrilling perceptions of violent crime (see Table 6, Model 3). While not of substantive interest, each demographic covariate was significant, such that being White or Hispanic (relative to Black) and having a parent with less education (a proxy for socioeconomic status) were associated with greater thrilling perceptions of violent crime (see Table 6, Model 4). In the full model, with all factors examined simultaneously, CU traits, peer delinquency, impulsive-irresponsible traits, and non-violent offending were the only main study variables significantly associated with the thrill of violent crime, such that higher scores on each were associated with increased thrill (see Table 6, Model 5).

**Table 6:** Mixed effect models estimating interaction terms between risk factors and age

	Thrill of Violent Crime			Thrill of Nonviolent Crime		
	*B*	*p*	*95% CI*	*B*	*p*	*95% CI*
Victimization X Age	0.025	0.526	−0.051, 0.100	0.002	0.882	−0.029, 0.034
Witnessing Violence X Age	0.017	0.314	−0.017, 0.051	−0.013	0.141	−0.029, 0.004
Neighborhood Disorder X Age	0.049	0.147	−0.017, 0.115	−0.016	0.426	−0.057, 0.024
Peer Delinquency X Age	0.031	0.453	−0.050, 0.112	−0.039	0.026	−0.074, −0.005
Impulsive-Irresponsible X Age	0.001	0.744	−0.006, 0.008	−0.004	0.077	−0.008, 0.000
Callous-Unemotional X Age	−0.005	0.276	−0.014, 0.004	−0.002	0.339	−0.008, 0.003
Grandiose-Manipulative X Age	−0.001	0.578	−0.006, 0.004	−0.001	0.510	−0.004, 0.002
Offending X Age	0.014	0.529	−0.030, 0.058	−0.006	0.239	−0.017, 0.004

Significant interaction terms between each risk factor and age were then added to the full model individually to test for any potential age interactions. No significant two-way interactions between the risk factors and age (see Table 4), indicating that the strength of the associations between the thrill of violent crime and these factors did not change across age.

## Discussion

Adolescents’ orientation towards rewards is consistently linked with their criminal behavior (Braams et al., 2015; Shulman et al., 2017; Thomas et al., 2020), but without much clarity as to the factors that foster or inhibit positive perceptions of criminal behavior. By simultaneously examining environmental and individual factors and distinguishing crime typology, the present study found that the thrills of violent and non-violent crime, respectively, are promoted by similar features of system-involved adolescents’ experiences environmentally, with distinct differences emerging among the individual factors tested.

### Environmental Factors

Our findings indicate that thrilling perceptions of any crime appear to be socially transmitted. Youths’ affiliation with peers who engage in delinquency was the only significant predictor among all the environmental characteristics evaluated. Prior research contends that youth partly shape their attitudes toward antisocial behavior from their peer groups (Albert et al., 2013). Peers facilitate expectations, behaviors, and perceptions of actions (Brechwald & Prinstein, 2011; Maxwell, 2002). Having peers who endorse crime as a thrilling experience may impress a similar orientation upon oneself. Assimilating to the attitudes of one’s friends is a hallmark of adolescence, given the desire to feel connected and included within one’s social environment (Warr & Stafford, 1991). This social transmission of thrilling perceptions of crime emphasizes the prominence of peer groups across adolescence and how adolescents may internalize values that mirror those of their friends.

Interestingly, none of the other tested environmental risk factors was significant. Notably, violence exposure did not predict thrilling perceptions of violent crime. One of the most consistently observed patterns in violence research is that those exposed to violence are much more likely to perpetrate the behavior (Benedini & Fagan, 2017; Mrug & Windle, 2010; Smith & Thornberry, 1995). The development of pro-criminal attitudes toward instrumental violence often perpetuates this cycle (Muradweij & Allwood, 2021; Alwood, 2008). The current study’s findings that these thrilling perceptions are not impacted by exposure to violence in the context of these other risk factors indicate that while violence exposure may promote perceptions of utility, the same pattern may not be true for thrilling perceptions. In other words, violence-exposed youth may be at risk for future violence, whether that be due to unconscious or conscious feelings of utility. Still, these findings suggest that they may not find violence thrilling merely because of that exposure.

### Individual Factors

Among the individual characteristics tested in the current study, the presence of callous-unemotional (CU) traits was found to be predictive of thrilling perceptions of violent criminal behavior. CU traits comprise the affective dimension of psychopathy characterized by a lack of empathy, shallow affect, and the absence of guilt. These features have been implicated in severe antisocial behavior in adolescents (Frick & White, 2008; Frick et al., 2014). Perhaps these traits foster thrilling perceptions of violent behavior, inadvertently leading to perpetuation. The lack of concern for others that CU traits confer may enable a sense of fun or excitement derived from behaviors inflicting harm. Physiological evaluations of youth with CU traits have observed blunted reactivity to emotional stimuli (Truedsson et al., 2019; Wagner & Waller, 2020). A recent review of extant literature by Northam and Dadds (2020) challenged this notion that all youth with these traits neglect to respond to emotionally driven events. These mixed findings suggest that these youth are less likely to respond to typical emotional stimuli than youth without these traits. Non-violent crimes are more common in adolescence and are perceived to be thrilling because they are less serious than violent crimes (Bensimon & Bodner, 2012). Youth with CU traits, however, may require more intense experiences to satiate that desire for thrill that is a hallmark of adolescence (Steinberg et al., 2017) but elevated in youth with CU traits (Frick & White, 2008; Frick et al., 2003). Furthermore, CU traits are also associated with greater fearlessness (Fanti et al., 2015), altogether placing these youth at risk for finding violent crime thrilling due to their callousness and elevated requirements to achieve that thrill from risky behavior. Research analyzing samples of system-involved youth has linked elevated levels of CU traits to violence (Muñoz & Frick, 2012), and these thrilling perceptions may be a potential mediator in this relationship.

As observed in this study, the impulsivity-irresponsible dimension of the YPI was associated with the thrill of both violent and non-violent crimes. These traits capture greater thrill-seeking tendencies and preferences for immediate satisfaction without regard for responsibilities. Current theories assert youth gradually develop the capacity to self-regulate via maturity and impulse control, which makes this time of heightened sensation seeking especially conducive to risk-taking (Gottfredson & Hirschi, 1990; Steinberg, 2008). The immaturity of these capacities may make risky scenarios seem more exciting than dangerous. Our finding that the grandiose-manipulative dimension of psychopathy was not associated with the thrill of either violent or non-violent crime, while unexpected, does align with Prospero-Luis et al.’s (2017) study investigating how psychopathic traits predict thrilling perceptions. Taken together, grandiose-manipulativeness may not influence thrilling perceptions of crime when examined amongst these other factors.

Consistent with prior research, greater offending, regardless of type, was associated with greater thrilling perceptions of any type of crime in our study. The more youth commit a crime, the more likely they find that crime thrilling. Youths’ experiences with these acts play a significant part in their perceptions of them. Importantly, offending was measured through self-report, meaning there may be crimes with which youth have never been criminally charged. Not getting caught for these behaviors may decrease crime deterrence, thereby eliciting a greater thrill given a successful experience. As Katz (1988) initially proposed, the allure of evading detection plays a significant role in thrilling perceptions of crime for adolescents. Additionally, this relationship may also be bidirectional. Finding crime thrilling, especially given adolescents’ greater orientation towards reward than punishment (Lee et al., 2018), makes them more likely to engage in it. In this context, it is difficult to disentangle whether the exposure to the act itself or merely its rewarding perception drives this relationship.

### Demographic Factors

Surprisingly, several demographic covariates were also uniquely linked with the thrill of crime. Socioeconomic status (SES), operationalized in this study as the highest level of education attained by a participant’s caregiver (Sirin, 2005), was negatively associated with the thrill of violent criminal behavior. This indicates that youth of lower SES are less likely to find violent criminal behavior thrilling. Interestingly, however, a meta-analysis by Piotrowska et al. (2015) concluded that SES and aggressive behavior are robustly associated. Future research may examine mechanisms that explain these incongruencies in perceptions and behaviors. Importantly, Piotrowska and colleagues parsed out the nuances of SES in an enriched way to genuinely capture how the dynamics of being socioeconomically disadvantaged facilitate criminal behavior. Capturing the role of perceptions of crime within in a similar fashion may provide a deeper understanding of how the experience of being of lower SES fosters these thrilling perceptions.

In terms of racial and ethnic identity, compared to Black-identifying youth, greater thrilling perceptions of violent and non-violent crime were observed for White- and Hispanic-identifying youth, respectively. While speculating is beyond the scope of this study, this association may be driven by baseline differences in key constructs a priori to these perceptions. Pederson and colleagues (2012) found that White youth exhibited higher and more pronounced growth in sensation seeking over time than Black youth, which may place White youth at greater risk for maladaptive thrilling perceptions in the context of these other risk factors included in the model. In appreciation of cultural differences within these populations, future research should take more detailed approaches to explain these race- and ethnicity-based differences in thrilling perceptions of criminal behavior.

### Age Interactions

In the present study, the only risk factor that varied as a function of age was the influence of peer delinquency on the thrill of non-violent crime, such that as participants aged, the influence of peer delinquency waned. This aligns with literature highlighting how youth are progressively more resistant to peer influence as they mature (Steinberg & Monahan, 2007; Sumter et al., 2009). Interestingly, prior findings suggest that peers who engage in delinquency contribute to non-violent criminal behavior more than violent criminal behavior (Bernburg & Thorlindsson, 1999). Therefore, as youth become more autonomous in their thinking, they may become less enticed by behaviors that may have initially only been driven by peer influence. Beyond peer influence on thrilling perceptions of non-violent crime, no other risk factor in the model differed as a function of age. While unexpected, these findings suggest that these risk factors confer risk for thrilling perceptions across adolescence. This was surprising given our restructuring of data by age to maximize variability, given that CU traits (Ray et al., 2019), impulsivity, and sensation seeking (Forrest et al., 2019) typically exhibit notable changes during adolescence rather than over time. The moderate stability of constructs such as CU traits, however, may make any age-related changes less impactful on their thrilling perceptions of crime (Takahashi et al., 2020). Ultimately, the lack of any other age-related interactions suggests that these factors confer risk for thrilling perceptions across adolescence. CU traits, for example, are considered notoriously difficult to treat (Wilkinson et al., 2015), and system-involved adolescents are not a treatment-seeking population (Yonek et al., 2019). Similarly, repeated offending may reinforce thrilling perceptions of the behavior, perpetuating a feedback loop that continues as adolescents age.

### Limitations

While this study provided an important perspective on the thrill of crime, there are limitations to consider. First, the current study examined a sample of system-involved youth who had committed serious crimes at recruitment; at least 94% had been charged with a felony offense. As such, it is difficult to generalize these findings to youth at the lower ends of the system or to youth not involved in the justice system. Thus, the generalizability of these findings to community adolescent samples is limited. However, as understanding the thrill of crime is best understood by those who engage in crime, the current sample provides an important step in understanding this mechanism.

Second, our sample was intentionally limited to youth aged 15–21 to assess the association between thrilling perceptions of crime and various risk factors during a developmental period where youth were most at risk for engaging in behaviors they consider rewarding (Geier, 2013). Experiences across the life course are crucial in forming and maintaining our perceptions of behaviors. Future studies might aim to evaluate these perceptions holistically across the lifespan and the various aspects of experience that shape them. While these risk factors were found to be salient in adolescence, certain experiences may vary in their impact across other life stages. More longitudinal investigations encompassing childhood, adolescence, and adulthood are critical to assessing how these perceptions fluctuate developmentally.

From a measurement perspective, items within the impulsive-irresponsible dimension of psychopathy (e.g., “I like to do things just for the thrill of it” or “I like to do exciting and dangerous things, even if it is forbidden or illegal”) inherently overlap with thrilling perceptions of crime, creating the potential for measurement redundancy. However, it was important to include some indicator of youths’ general propensity for sensation seeking to distinguish it from crime-specific thrilling perceptions while also accounting for its contributions to the development of these perceptions. Given that these constructs have traditionally been viewed as two sides of the same coin, future research should be more deliberate in disentangling general sensation seeking from the specific behaviors that youth derive pleasure from.

Finally, while it may be that engaging with peers who commit delinquent behavior facilitates thrilling perceptions, the measure neglects to inquire about the peers’ perceptions of these behaviors. Participants were not explicitly asked why they believe their peers engaged in these behaviors, so it is unclear whether the hypothesized social contagion effect occurs. However, research suggests that peer behavior is more impactful than peer attitudes on criminal behavior (Warr & Stafford, 1991). Future research may also seek to evaluate potential mediating pathways to explain certain findings, such as neighborhood disorder, for example, whose effects were initially significant but dissipated in the final model. It would be worthwhile for future studies to explore potential indirect relationships between these predictors and their association with thrilling perceptions of crime.

Despite these limitations, the current study simultaneously examined several potential factors that promote thrilling perceptions of criminal behavior. While a variety of studies have leveraged data from the Pathways to Desistance Study to examine perceived rewards of crime, those studies have primarily focused on how such perceptions influence adolescents’ offending within rational choice frameworks in addition to age-related considerations. In contrast, the current study parses thrilling perceptions of violent and non-violent crime and builds on prior work by integrating known predictors to comprehensively assess how individual and environmental factors in system-involved adolescents’ lives shape the development of these perceptions. Utilizing this ecological approach highlighted influences at various layers of an adolescents' context. While their offending behavior and impulsive-irresponsibility predicted greater thrilling perceptions of crime, they were similarly impacted by peers engaging in delinquency. These individual differences, paired with these socially transmitted beliefs from peers, highlighted how thrilling perceptions of crime may be reinforced by their own experiences or vicariously through their peers. Leveraging the salience of adolescents’ impulsivity and susceptibility to peer influence may curb these criminogenic perceptions by orienting youth towards healthier forms of risk-taking (Duell & Steinberg, 2018). Recreational sports, for example, provide the opportunity to satiate the desire for thrills in a socially acceptable way (Hansen & Breivik, 2001), fostering prosocial bonds through team-based activities with non-delinquency engaging peers (Bruner et al., 2017).

Thrilling perceptions of violent crime were uniquely predicted by CU traits, which have been linked with violent behavior (Frick & White, 2008; Frick et al., 2014; Muñoz & Frick, 2012). These perceptions may preclude engagement in violent crime. Thus, future investigations should examine the mediating role of these perceptions. Additionally, interventions promoting emotional processing and interpersonal skills targeting the deficits associated with CU traits (Wilkinson et al., 2015) may reduce these traits and, by proxy, these criminogenic perceptions. This study’s findings highlight how pivotal adolescents’ characteristics and those of their peers are to perceiving crime as thrilling, requiring similarly holistic approaches to reduce these perceptions.
